# Claudin 24—A novel enhancer of AMPA receptor fidelity

**DOI:** 10.1126/sciadv.aeb0196

**Published:** 2026-03-06

**Authors:** Tobias Strasdeit, Ehsan Amin, Sebastian Obst, Barbara Biermann, Thomas P. Newton, Simon Chris Kösters, Marten Schouwink, Sergey Fedotov, Javeria Shaukat, Subhrajit Bhattacharya, Muhammad Aslam, Max Anstötz, Faik Nadi Okka, Oscar Gabriel Sevillano Quispe, Pascal Bouvain, Julia Vedyashkin, Alexander I. Sobolevsky, Stephen F. Traynelis, Jakob von Engelhardt, Nadine Erlenhardt, Michael Hollmann, Nikolaj Klöcker

**Affiliations:** ^1^ Institute of Neuro- and Sensory Physiology, Medical Faculty, Heinrich Heine University, University Hospital Düsseldorf, 40225 Düsseldorf, Germany.; ^2^ Department of Biochemistry I—Receptor Biochemistry, Ruhr University Bochum, 44780 Bochum, Germany.; ^3^ Light Microscopy Technology Platform (LiMiTec), Medical School OWL, Bielefeld University, 33615 Bielefeld, Germany.; ^4^ Department of Biochemistry and Molecular Biophysics, Columbia University, New York, NY 10032, USA.; ^5^ Cellular, Molecular, and Biomedical Studies Umbrella Program, Columbia University Irving Medical Center, New York, NY 10032, USA.; ^6^ Department of Pharmacology and Chemical Biology, Emory University School of Medicine, Atlanta, GA 30322, USA.; ^7^ School of Pharmacy, Keck Graduate Institute, The Claremont Colleges, Claremont, CA 91711, USA.; ^8^ Institute of Pathophysiology, Focus Program Translational Neuroscience (FTN), University Medical Center of the Johannes Gutenberg University Mainz, 55128 Mainz, Germany.; ^9^ Institute of Anatomy II, Medical Faculty, Heinrich Heine University, University Hospital Düsseldorf, 40225 Düsseldorf, Germany.; ^10^ Experimental Cardiovascular Imaging, Institute of Molecular Cardiology, Medical Faculty, Heinrich Heine University, University Hospital Düsseldorf, 40225 Düsseldorf, Germany.

## Abstract

High-fidelity fast excitatory neurotransmission in the mammalian central nervous system is conducted by the AMPA receptor (AMPAR) subfamily of ionotropic glutamate receptors. AMPARs exist as complexes of the pore-lining α subunits GluA1–4 and a number of auxiliary subunits shaping their functional properties. The first discovered family of auxiliary subunits comprises the transmembrane AMPAR regulatory proteins (TARPs). Together with germ cell–specific gene 1–like (GSG1L), they belong to the PMP-22/EMP/MP20/claudin superfamily sharing substantial sequence homology and a 4-transmembrane domain (4-TMD) topology. Here, we identify the claudin Cldn24 as a novel AMPAR auxiliary subunit in cerebellar granule cells (CGCs). Specifically accelerating the recovery of desensitized receptors, Cldn24 counterbalances gating modulation of GluA by TARP-γ2 and GSG1L in CGCs and hence enables fast reactivation of native receptors. These features substantially expand the tuning properties of hitherto known AMPAR auxiliary subunits and render Cldn24 a powerful enhancer of AMPAR fidelity.

## Teaser

The claudin subfamily family member Cldn24 is identified as an AMPAR auxiliary subunit.

## INTRODUCTION

Postsynaptic glutamate receptors of the AMPA subtype (AMPARs) mediate fast signal transmission at excitatory synapses in the central nervous system (CNS) in response to synaptic release of glutamate (*1*). In addition, synaptic glutamate also binds to and activates the *N*-methyl-d-aspartate (NMDA) subtype of glutamate receptors, which promotes downstream signaling pathways that can mediate synaptic plasticity (*2*, *3*). The AMPAR response time course varies greatly between synapses with respect to developmental stage and CNS region. Their impressive functional diversity is achieved by differential assembly of the four pore-lining subunits GluA1–4, their alternative splicing, RNA editing, and by posttranslational modification (*4*–*6*). Moreover, several transiently interacting proteins and a number of coassembling auxiliary subunits regulate subcellular trafficking and distribution of AMPARs as well as shape their gating properties (*7*–*11*). By definition, auxiliary subunits do not form ion pores themselves but associate with the receptor along the secretory pathway and thus become constituents of surface AMPAR complexes, where they modulate AMPAR function in ways that can be detected both in vivo and in vitro (*8*, *12*). To date, five protein families fulfill the criteria for AMPAR auxiliary subunits: transmembrane AMPAR regulatory proteins (TARPs), protein cornichon homologs (CNIHs), germ cell–specific gene 1–like (GSG1L), cystine-knot AMPA receptor modulating proteins (CKAMPs/Shisas), and proline-rich transmembrane protein 1 (PRRT1/SynDIG4) (*1*, *11*). TARPs and GSG1L share a characteristic 4-transmembrane domain (4-TMD) topology with the claudin superfamily of proteins.

Claudins comprise a large family of integral tight junction proteins, central to forming epithelial barriers or ion-selective openings (*13*). They are known to assemble as homo- or heterotypic multimers, both in cis orientation along the plasma membrane of a single cell and in trans orientation across adjacent cells. For the latter, they critically rely on their extracellular loops, with the first loop defining ion selectivity of paracellular transport and the second one mediating trans interaction (*14*). The most prominent claudin members in the brain include Cldn5, sealing the blood-brain barrier (*15*), and Cldn11, serving myelin integrity (*16*). For other members, including Cldn20–27, either ambiguous or no data exist on their physiological role, challenging their classification as claudins (*13*, *17*, *18*). As the TARP-γ2 shares characteristic claudin-like functions in causing mouse fibroblasts to adhere to each other (*19*), we wondered whether claudins, in turn, may share TARP-like properties in AMPAR modulation.

## RESULTS

### Identification of claudins as modulators of GluA receptors

**Fig. 1.**
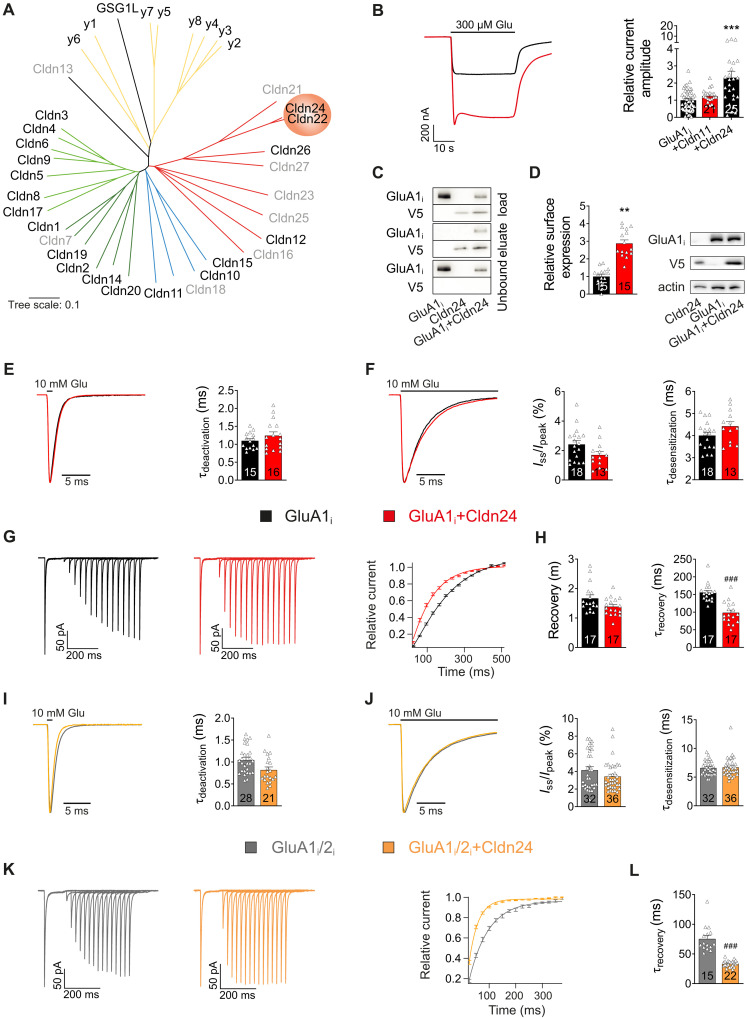
Cldn24 assembles with GluA and modulates its gating. (**A**) Phylogenetic tree of TARPs, GSG1L, and claudins (scale: 0.1 amino acid substitutions per amino acid). (**B**) Glutamate-induced GluA1 current (*N* = 48, SEM = 0.09; TEVC) was significantly augmented by Cldn24 (2.31 ± 0.37-fold, *N* = 25, *P* = 0.0003), but not by Cldn11 (1.24 ± 0.09-fold, *N* = 21, *P* = 0.3079). (**C**) Coimmunoprecipitation of GluA1 and V5-tagged Cldn24. (**D**) The amount of GluA1 on the cell surface (SEM = 0.13, *N* = 15; extracellular epitope tagging) was increased by Cldn24 (2.88 ± 0.20, *N* = 15, *P* < 0.0001). (**E** to **H**) Outside-out patch recordings of GluA1. The τ_deactivation_ (1.10 ± 0.06 ms, *n* = 15, *P* = 0.4945), current ratio *I*_ss_/*I*_peak_ (2.42 ± 0.26%, *n* = 18), and τ_desensitization_ (4.00 ± 0.15 ms, *n* = 18) of GluA1 remained unchanged by Cldn24 (1.24 ± 0.10 ms, *n* = 16; 1.70 ± 0.24%, *n* = 13, *P* = 0.0681; 4.42 ± 0.20 ms, *n* = 13, *P* = 0.1056). However, recovery from desensitization (155 ± 6.28 ms, *n* = 17) was significantly accelerated by Cldn24 (98.3 ± 7.50 ms, *n* = 17, *P* < 0.0001). Fitting recovery profiles in (G) by monoexponential Hodgkin-Huxley equations revealed no change in *m* value (GluA1: 1.67 ± 0.12, *n* = 17; GluA1 + Cldn24: 1.39 ± 0.07, *n* = 17, *P* = 0.0851). (**I** to **L**) GluA1/2 recordings as in (E) to (H). The τ_deactivation_ of GluA1/2 (1.04 ± 0.06 ms, *n* = 28), *I*_ss_/*I*_peak_ (4.15 ± 0.38%, *n* = 32), and τ_desensitization_ (6.60 ± 0.21 ms, *n* = 32) remained unaltered by Cldn24 (0.82 ± 0.07 ms, *n* = 21, *P* = 0.0148; 3.27 ± 0.23%, *n* = 35, *P* = 0.1952; 6.51 ± 0.20 ms, *n* = 35, *P* = 0.8370). Recovery of GluA1/2 from desensitization (75.5 ± 5.72 ms, *n* = 15) was significantly accelerated by Cldn24 (33.5 ± 1.17 ms, *n* = 22, *P* < 0.0001). Recovery profiles in (K) were fitted monoexponentially. Data in (B) were analyzed by Kruskal-Wallis test following Dunn’s multiple comparisons with control and data in (D) by unpaired *t* test. **P < 0.01; ***P < 0.001. Data in (E), (F), and (H) (^###^*P* < 0.0002) and (I), (J), and (L) (^###^*P* < 0.00025) were analyzed by Mann-Whitney *U* test and corrected for family-wise error.

Led by the 4-TMD topology shared with prominent AMPAR auxiliary subunits such as TARPs and GSG1L, we screened the claudin family of proteins for potential effects on GluA receptor currents. We used two-electrode voltage clamp (TEVC) recording of glutamate-mediated (300 μM) currents from *Xenopus laevis* oocytes coexpressing claudins with homomeric GluA1 or heteromeric GluA1/2 receptors, each as flip variant. Of all claudins depicted in Fig. 1A, most of which could be successfully cloned from rat brain, Cldn22 and Cldn24 were identified as interesting candidates for being AMPAR modulators. They approximately doubled steady-state current amplitudes of both GluA1i homomers and GluA1i/2i heteromers (Fig. 1B and fig. S1, A and B). As Cldn22 and Cldn24 share >90% primary sequence homology, with Cldn24 being slightly more effective in potentiating GluA steady-state currents, we focused on the latter for further analysis. The desensitization inhibitor trichloromethiazide (TCM; 600 μM) only partially reduced the observed GluA current amplification (fig. S1A), indicating a rather complex mechanism of claudin action on GluA, with at least one component being independent of receptor desensitization. Hence, we tested whether coexpression of Cldn24 was able to modulate the amount of GluA protein on the cell surface using an extracellular epitope tagging approach. Cldn24 indeed increased surface expression of extracellularly hemagglutinin (HA)–tagged GluA1 [GluA1(HAex)] in *X. laevis* oocytes by a factor of 2.88 ± 0.20 (*N* = 15 oocytes, *P* < 0.0001), whereas total GluA1 expression remained unaffected by coexpression of Cldn24 (Fig. 1D).

Given the lack of a Cldn24-specific anti-claudin antibody, we checked for integration of Cldn24 into GluA1 protein complexes in recombinant coimmunoprecipitation experiments using artificial epitope tagging. Figure 1C shows that GluA1 was copurified by anti-V5 from membrane fractions of *X. laevis* oocytes coexpressing GluA1 and V5-tagged Cldn24, confirming their molecular assembly. Known to be ubiquitously expressed in brain epithelia (*20*), Cldn11 provided a convenient negative control, binding to GluA1 but not functionally modulating its currents (fig. S1, H to L). Of note, coexpression of Cldn24 with other ionotropic glutamate receptors, i.e., NMDA and kainic acid (KA) receptors, did not affect their steady-state current.

### Cldn24 accelerates the recovery of GluA receptors from desensitization

Potential effects of claudin assembly with GluA1 on receptor gating were investigated in detail using piezo-controlled fast application of glutamate (10 mM) onto outside-out patches from *X. laevis* oocytes. Exhibiting prominent effects on steady-state currents in TEVC mode, Cldn24 altered neither deactivation nor desensitization kinetics of both GluA1 homomers and GluA1/2 heteromers (Fig. 1, E, F, I and J). However, Cldn24 markedly accelerated the recovery of receptors from desensitization in both GluA1 homomers and GluA1/2 heteromers (Fig. 1, G and K). As quantified in Fig. 1, H and L, respectively, Cldn24 reduced the time constant of GluA1 recovery from desensitization (τ_recovery_) from 155 ± 6.28 ms (*n* = 17) to 98.3 ± 7.50 ms (*n* = 17, *P* < 0.0001) and from 75.5 ± 5.72 ms (*n* = 15) to 33.5 ± 1.17 ms (*n* = 22, *P* < 0.0001) in GluA1/2 heteromers. Unexpectedly, the GluA1 and GluA1/2 steady state–to–peak current response ratio did not increase by coassembly with Cldn24 (Fig. 1, F and J). This unusual finding may at least in part be plausibilized by our noise analysis revealing that Cldn24 reduced the mean single-channel conductance of GluA1 homomers from 24.5 to 14.2 pS, leaving channel open probability unaffected (fig. S1, E to G).

**Fig. 2.**
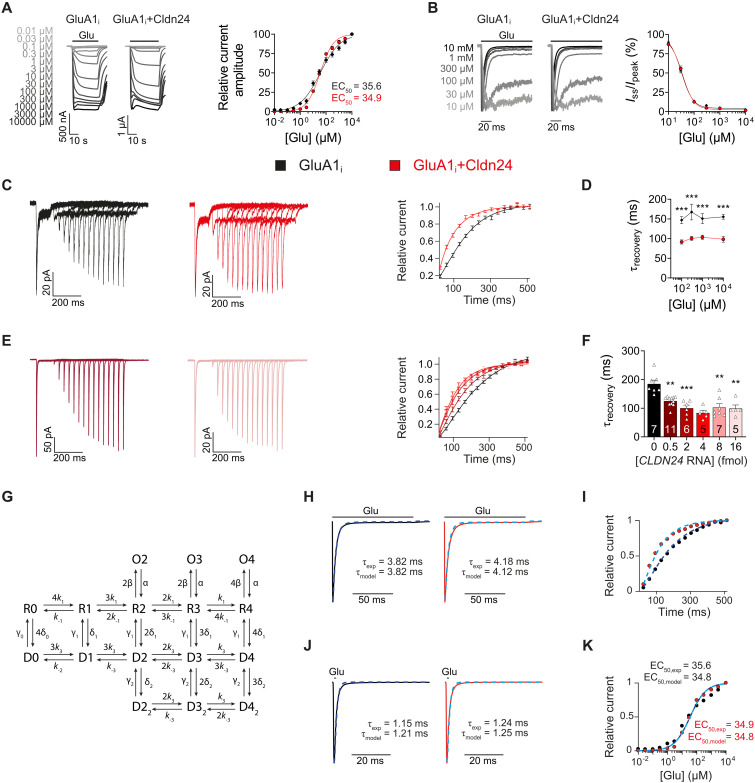
Cldn24 specifically accelerates GluA1 recovery from desensitization. (**A**) TEVC-recorded currents at indicated glutamate concentrations. Hill equation fits of concentration-response of GluA1 (*n* = 14) and GluA1 + Cldn24 (*n* = 16), yielding EC_50_ values of 35.6 and 34.9 μM, respectively. (**B**) Outside-out patch recordings of GluA1. *I*_ss_/*I*_peak_ (in percent) current upon 100-ms applications of indicated glutamate concentrations. (**C** and **D**) Recovery of GluA1 at indicated glutamate concentrations. At 100 μM glutamate (C), τ_recovery_ decreased from 147 ± 9.14 ms (*n* = 15) to 91.5 ± 5.31 ms (*n* = 19, *P* < 0.0001), at 300 μM glutamate from 167 ± 20.0 ms (*n* = 10) to 101 ± 4.89 ms (*n* = 13, *P* = 0.0002), at 1 mM glutamate from 151 ± 13.0 ms (*n* = 9) to 103 ± 5.40 ms (*n* = 13, *P* = 0.0002) and at 10 mM glutamate from 155 ± 6.28 ms (*n* = 17) to 98.3 ± 7.50 ms (*n* = 17, *P* < 0.0001). (**E** and **F**) Recovery of GluA1 coexpressed with different amounts of Cldn24. Recovery of GluA1 (185 ± 13.2 ms, *n* = 7) was significantly accelerated by 2 fmol (100 ± 10.7 ms, *n* = 6, *P* = 0.0040), 4 fmol (83.5 ± 9.08 ms, *n* = 5, *P* < 0.0002), 8 fmol (103 ± 12.7 ms, *n* = 7, *P* = 0.0030), and 16 fmol [99.1 ± 12.1 ms, *n* = 5, *P* = 0.0075 (E), right)], but not significantly by 0.5 fmol of coinjected Cldn24 cRNA [125 ± 5.06 ms, *n* = 11, *P* = 0.0991 (E), left)]. (**G**) Kinetic scheme for GluA1, modified from previously published schemes (*21*, *22*). Conductances for the three open states (O2 to O4) were set to 9, 15, and 21 pS. (**H** to **K**) Experimental data, overlaid with simulated responses (dashed blue lines). Simulated parameters are close to the experimental data (GluA1: τ_desensitization_ = 3.82 ms, χ^2^ = 4.19 × 10^−6^; τ_deactivation_ = 1.21 ms, χ^2^ = 3.13 × 10^−3^; EC_50_ = 34.8 μM, χ^2^ = 1.80 × 10^−2^; GluA1 + Cldn24: τ_desensitization_ = 4.12 ms, χ^2^ = 8.61 × 10^−4^; τ_deactivation_ = 1.25 ms, χ^2^ = 8.06 × 10^−5^; EC_50_ = 34.8 μM, χ^2^ = 2.87 × 10^−4^). In addition, simulated recovery time course fits the experimental data closely (GluA1: χ^2^ = 1.03 × 10^−3^; GluA1 + Cldn24: χ^2^ = 1.09 × 10^−3^). Data were analyzed by Mann-Whitney *U* test (D) or Kruskal-Wallis test following Dunn’s multiple comparisons with control (F). **P < 0.01; ***P < 0.001.

As the other 4-TMD AMPAR auxiliary subunits change the apparent ligand affinities of GluAs (*1*), concentration-response profiles of GluA1 were obtained in the absence and presence of Cldn24. Figure 2 (A and B) shows that neither the median effective concentration (EC_50_) determined by TEVC recording of steady-state currents nor the concentration dependence of the steady state–to–peak current ratio determined in outside-out patch clamp recordings was changed by coexpression of Cldn24. For all ligand concentrations tested, the characteristic acceleration of the recovery of GluA1 from desensitization by Cldn24 was in a similar range (Fig. 2D). Neither agonist binding properties of GluA1 probed by the glutamate/kainate ratio were apparently modulated by Cldn24 nor were its current-voltage relationship and rectification properties (fig. S2, A and B).

Reducing the amount of Cldn24 available for GluA1 receptor coassembly by decreasing the ratio of injected complementary RNAs (cRNAs) coding for Cldn24 and GluA1 from 1:2 to 1:16 also tended to reduce the acceleration of GluA1 recovery from desensitization (τ_recovery_ = 125 ± 5.06 ms, *n* = 11, *P* = 0.0991; Fig. 2, E and F). For ratios of 1:4 up to 2:1, τ_recovery_ remained significantly accelerated over control without further change, indicating that the standard conditions used in our experiments, a cRNA ratio of Cldn24 to GluA1 of 1:2, gave rise to GluA complexes fully saturated with Cldn24.

### Mathematical modeling of GluA gating modulation by Cldn24

To better understand how Cldn24 affects GluA gating, we modified a previously reported mathematical model of GluA1 gating to take into account our experimental findings (*21*, *22*). When comparing our experimental data with simulations by the model recently developed by Coombs *et al.* (*21*), we noticed that in contrast to the modeled desensitization rates, the ones for deactivation were far slower than experimentally observed. This discrepancy suggests that the kinetics of ligand dissociation from the “R” states may differ from the ones from the “D” states. As shown in Fig. 2G, we therefore introduced distinct rate constants for the liganded “D” states, represented as *k*_3_/*k*_−3_. Our refined mathematical model fits our experimental data on GluA1 gating, both with and without Cldn24. We were able to accurately reproduce all measured parameters for deactivation, desensitization, dose-dependent steady-state current, and recovery kinetics (Fig. 2, H to K). χ^2^ values (χ^2^_GluA1_ = 1.34 × 10^−1^, χ^2^_GluA1 + C24_ = 4.20 × 10^−3^) served as indicators of how well the modified model aligned with experimental results. The relevant kinetic parameters and their alterations by the presence of Cldn24 are summarized in Table 1. The most notable changes induced by Cldn24 shifts the gating equilibrium in favor of faster recovery of GluA1, predominantly from its deep desensitization to its resting states, eventually increasing chances for receptor reactivation (fig. S2C and movie S1).

**Table 1.** Rate constants for the gating scheme in Fig. 2. Rate constants obtained from fitting the model to the respective experimental data. Relative changes in rate constants caused by Cldn24 coexpression are given in percent.

	α (s^−1^)	β (s^−1^)	δ_0_ (s^−1^)	ϒ_0_ (s^−1^)	δ_1_ (s^−1^)	ϒ_1_ (s^−1^)	δ_2_ (s^−1^)	ϒ_2_ (s^−1^)	*k*_1_ (M^−1^ s^−1^)	*k*_−1_ (s^−1^)	*k*_−2_ (s^−1^)	*k*_3_ (s^−1^)	*k*_−3_ (s^−1^)
GluA1	3618	13164	0.17	23.6	1126	7.43	20.1	22.8	5.01E + 07	6275	0.02	1.31E + 06	164
GluA1 + Cldn24	1873	7464	0.19	39.3	1263	11.7	48.9	48.8	6.94E + 07	9646	0.02	1.26E + 06	175
Change	−48%	−43%	11%	67%	12%	58%	144%	114%	38%	54%	0%	−4%	7%

### Cldn24 is an AMPAR auxiliary subunit in cerebellar granule cells

**Fig. 3.**
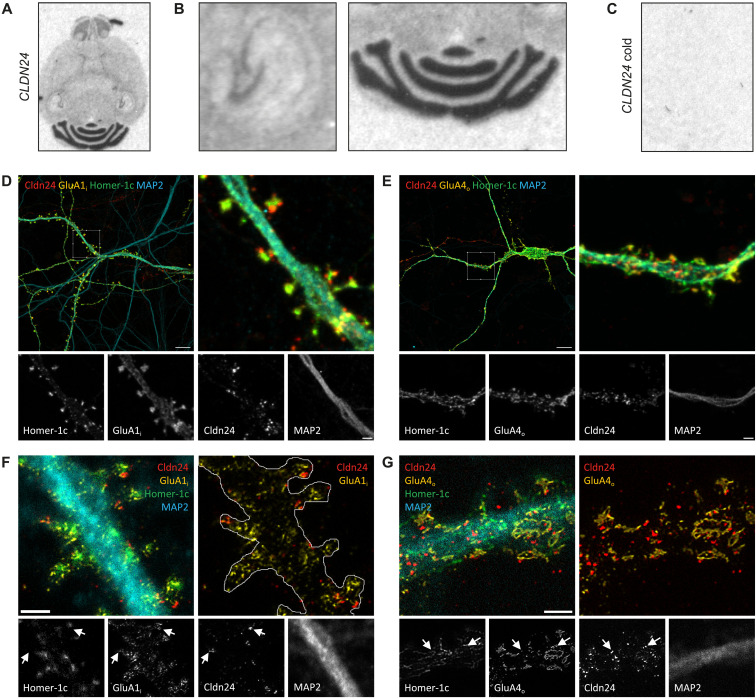
Cldn24 colocalizes with synaptic GluAs in both hippocampal and cerebellar neurons. (**A**) Expression of Cldn24 mRNA in adult mouse brain was revealed by in situ hybridization. Autoradiographic images of 15-μm brain slices hybridized with an oligodeoxyribonucleotide Cldn24 probe (5′-ATGGCTTTCATCTTCAGAAC GGCCATGCAATCAGTAGGGCTTTCT-3′). (**B**) Magnified image of (A) of the hippocampus (left) and the cerebellum. (**C**) Control hybridization with a 10× excess of unlabeled (cold) Cldn24 probe demonstrating specificity of the hybridization signal by competitive inhibition. (**D**) Immunostaining of cultured hippocampal neurons [21 days in vitro (DIV21)] transduced with GluA1(HA-ex), Cldn24(Myc-ex), and Homer-1c-EGFP. Representative confocal image of dendrites of a hippocampal neuron, quadruple-labeled as a merge of all signals as an overview and zoomed in. Separately imaged signals for Homer-1c, GluA1, and Cldn24. Endogenously expressed microtubule-associated protein 2 (MAP2) served as a dendritic marker. Scale bars, 10 μm (top); 2 μm (bottom). Pixel size: 113.6 nm by 113.6 nm. (**E**) Immunostaining of cultured CGCs (DIV14) transduced with GluA4(HA-ex), Cldn24(Myc-ex), and Homer-1c-EGFP. Representative confocal image of dendrites of a granule cell. Scale bars, 10 μm (top); 2 μm (bottom). Pixel size: 113.6 nm by 113.6 nm. (**F**) Representative stimulated emission depletion (STED) image of a dendrite of a hippocampal neuron with several spines, quadruple-labeled as a merge of all signals without and with estimated shape of spines. Separately imaged signals for Cldn24, GluA1, Homer-1c, and MAP2 (the latter confocal without STED). Arrows indicate colocalization. Scale bar, 1 μm. Pixel size: 19.3 nm by 19.3 nm. (**G**) Representative STED image of a dendrite of a granule cell. Scale bar, 1 μm. Pixel size: 19.95 nm by 19.95 nm.

Having recognized and validated Cldn24 as a modulator of GluA trafficking and gating in a recombinant expression system, we next addressed the question of whether Cldn24 may serve as an auxiliary subunit in native AMPARs. In situ hybridization revealed circumscribed mRNA expression of Cldn24 in mouse brain (Fig. 3A). In particular, the dentate gyrus and cornu ammonis 1 (CA1) region of the hippocampus and, to greater extent, cerebellar granule cells (CGCs) stained positive for Cldn24 mRNA. Probe specificity was validated by competitive hybridization, in which signal was abolished upon coincubation with an excess of unlabeled Cldn24 probe (Fig. 3C). Due to the absence of Cldn24-specific antibodies, we used epitope tagging of Cldn24 to investigate its subcellular localization in dissociated and transfected murine hippocampal neurons and CGCs. Insertion of a Myc-tag into the second extracellular loop of Cldn24 [Cldn24(Myc-ex)] did not impair its functional effects on GluA1 homomeric receptors as confirmed by outside-out patch clamp recordings (fig. S3, B and C), but allowed for detecting Cldn24 protein expression on the cell surface. Extracellularly Myc-tagged Cldn24 was found to closely colocalize with HA-tagged GluA1 and HA-tagged GluA4 in postsynaptic membranes of transduced hippocampal neurons and CGCs, respectively, compatible with physical interaction (Fig. 3, D to G).

**Fig. 4.**
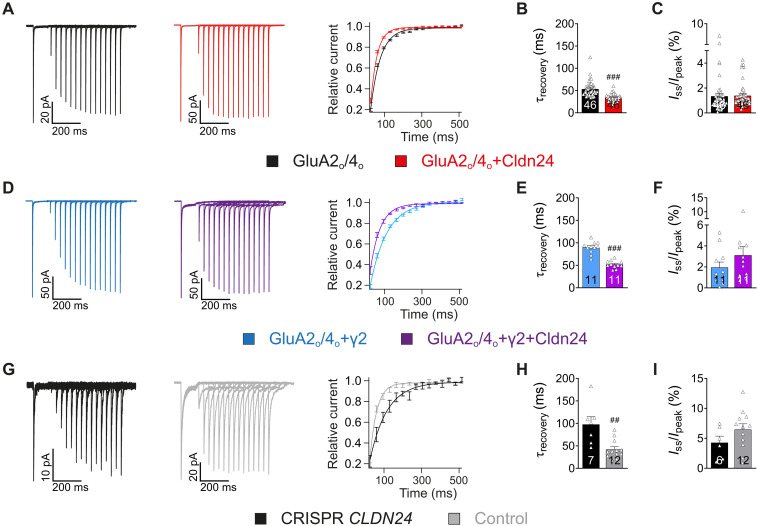
Cldn24 speeds up the recovery from desensitization of AMPARs in CGCs. (**A**) Recovery of GluA2/4 with and without Cldn24. Recordings as in Fig. 1G. (**B**) Recovery of GluA2/4 (τ_recovery_ = 53.5 ± 2.36 ms, *n* = 46) was significantly accelerated by Cldn24 (τ_recovery_ = 34.0 ± 1.10 ms, *n* = 46, *P* < 0.0001). (**C**) The current ratio *I*_ss_/*I*_peak_ of GluA2/4 (1.34 ± 0.22%, *n* = 46) remained unaltered by Cldn24 (1.39 ± 0.15%, *n* = 46, *P* = 0.1641). (**D**) Recovery of GluA2/4 coexpressed with TARP-γ2 with and without Cldn24. (**E**) Recovery of GluA2/4 + γ2 (τ_recovery_ = 89.7 ± 4.04 ms, *n* = 11) was significantly accelerated by Cldn24 (τ_recovery_ = 52.0 ± 2.54 ms, *n* = 11, *P* < 0.0001). (**F**) The current ratio *I*_ss_/*I*_peak_ of GluA2/4 + γ2 (1.95 ± 0.51%, *n* = 11) tended to be increased by Cldn24 (3.11 ± 0.81%, *n* = 11, *P* = 0.2703). (**G**) Recovery of AMPARs from steady-state desensitization in outside-out patches of CGCs of either native mice or after CRISPR-Cas9–mediated knockout of Cldn24. (**H**) Recovery of AMPARs in native granule cells (τ_recovery_ = 42.1 ± 6.02 ms, *n* = 12) was significantly slower when CRISPR-Cas9 was used to knock out Cldn24 (τ_recovery_ = 97.6 ± 17.2 ms, *n* = 7, *P* = 0.0018). (**I**) The current ratio *I*_ss_/*I*_peak_ of AMPARs in CGCs (6.47 ± 0.96%, *n* = 12) tended to decrease after CRISPR-Cas9–mediated knockout of Cldn24 (4.28 ± 1.08%, *n* = 7, *P* = 0.1800). Data were analyzed by Mann-Whitney *U* test and corrected for family-wise error. ^##^*P* < 0.005; ^###^*P* < 0.0005.

CGCs are the postsynaptic target of the high-frequency mossy fiber projection into the cerebellum. Besides GluA1, CGCs express predominantly the fast-gating GluA2 and GluA4 subunits as alternatively spliced flop variants (*23*, *24*). Probing heteromeric GluA2/4 as flop variants in *X. laevis* oocytes revealed that recovery from desensitization of these fast-gating GluA heteromers is accelerated by Cldn24 (Fig. 4A). The time constant τ_recovery_ decreased from 53.5 ± 2.36 ms to 34.0 ± 1.10 ms for GluA2/4 in the absence (*n* = 46) and presence of Cldn24, respectively (*n* = 46, *P* < 0.0001; Fig. 4B). Encouraged by these results, we performed outside-out patch clamp recordings from cultured CGCs, in which Cldn24 was knocked out by CRISPR-Cas9 technology. In agreement with a relevant role of Cldn24 as an auxiliary subunit in cerebellar AMPARs, its knockout significantly slowed the recovery of AMPAR currents from desensitization with τ_recovery_ increasing from 42.1 ± 6.02 ms (*n* = 12) in wild-type CGCs to 97.6 ± 17.2 ms in Cldn24 knockout CGCs (*n* = 7, *P* = 0.0018; Fig. 4, G and H).

Still, native AMPAR gating kinetics in CGCs did not fully match the ones of heterologously expressed GluA2/4-Cldn24 complexes. In an attempt to reconstitute the native situation more closely, we additionally coexpressed the TARP-γ2, a prerequisite for surface localization of AMPARs in CGCs (*25*). As shown in fig. S4G and previously reported by Miguez-Cabello *et al.* (*26*), the TARP-γ2 by itself slowed the recovery of GluA2-containing receptors from desensitization (τ_recovery_ = 89.7 ± 4.04 ms, *n* = 11, *P* = 0.0002), whereas adding Cldn24 effectively antagonized the delay and reduced τ_recovery_ to 52.0 ± 2.54 ms (*n* = 11, *P* < 0.0001; Fig. 4, D and E). Tripartite complex formation was confirmed by heterologous coimmunoprecipitation experiments (fig. S4I). Comparable results were obtained with GSG1L readily expressed in CGCs and well known to retard GluA recovery from desensitization (fig. S4). Cldn24 was able to significantly increase the nondesensitizing current component [*I*_ss_/*I*_peak_ (in percent)] of GluA2/4 (1.35 ± 0.22%, *n* = 46), when either TARP-γ2 (3.12 ± 0.81%, *n* = 11, *P* = 0.0053), or GSG1L (2.53 ± 0.50%, *n* = 20, *P* = 0.0042), or both were present (3.56 ± 0.64%, *n* = 20 *P* < 0.0001; fig. S4H).

**Fig. 5.**
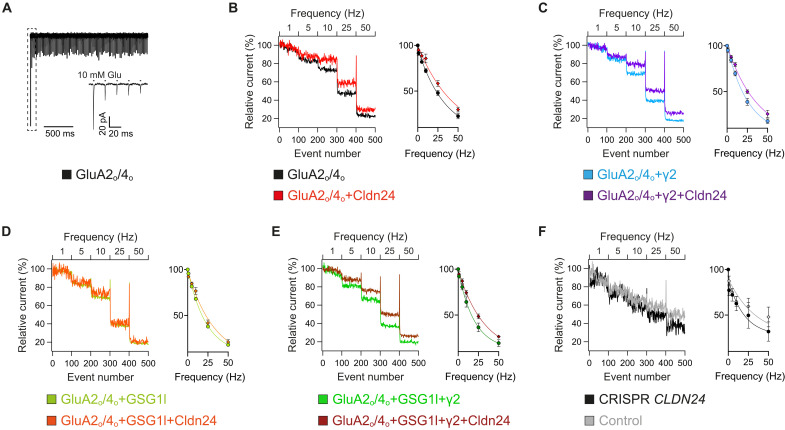
Cldn24 enables high-frequent reactivation of AMPARs. (**A**) Responses of GluA2/4 upon a 50-Hz train of 1-ms applications of glutamate, and scale-up of the first five responses (inset). (**B**) Pooled data of relative peak current trains of GluA2/4 with and without Cldn24 at indicated stimulus frequencies, normalized to the peak current of the first response in each case [*I*/*I*_0_ (in percent)]. Averaged data points of the last 20 values of each frequency train fitted monoexponentially (in percent). Frequency-dependent signal depression τ of GluA2/4 (31.5 ± 3.44 Hz, *n* = 8) tended to be increased by Cldn24 (42.2 ± 4.45 Hz, *n* = 6, *P* = 0.1079). (**C**) GluA2/4 coexpressed with γ2. Frequency-dependent signal depression τ of GluA2/4 + γ2 (26.9 ± 2.82 Hz, *n* = 8) tended to be increased by Cldn24 (34.4 ± 2.55 Hz, *n* = 5, *P* = 0.0932). (**D**) GluA2/4 coexpressed with GSG1L. Frequency-dependent signal depression τ of GluA2/4 + GSG1L (23.3 ± 1.63 Hz, *n* = 11) tended to be increased by Cldn24 (29.8 ± 3.98 Hz, *n* = 4, *P* = 0.1040). (**E**) GluA2/4 coexpressed with GSG1L + γ2. Frequency-dependent signal depression τ of GluA2/4 + GSG1L + γ2 (24.4 ± 5.15 Hz, *n* = 6) tended to be increased by Cldn24 (31.2 ± 3.39 Hz, *n* = 9, *P* = 0.2238). (**F**) Native granule cells or after CRISPR-Cas9–mediated knockout of Cldn24. Frequency-dependent signal depression τ of AMPARs in CGCs (43.8 ± 15.6 Hz, *n* = 3) tended to be lower when CRISPR-Cas9 was used to knock out Cldn24 (26.7 ± 5.73 Hz, *n* = 3, *P* = 0.7000). Data were analyzed by Mann-Whitney *U* test.

To estimate the putative impact of Cldn24 on postsynaptic AMPAR signaling, we applied trains of 1-ms pulses of saturating ligand to heteromeric GluA2/4 alone or in combination with TARP-γ2 and/or GSG1L and quantified peak currents at increasing stimulation frequencies upon coexpression of Cldn24 (Fig. 5, A to E). Consistently, frequency-dependent signal depression was less pronounced when Cldn24 was coexpressed. The same held true for native AMPAR currents recorded from CGCs exhibiting more severe frequency-dependent signal depression when Cldn24 was knocked out (Fig. 5F).

### Structure prediction of the Cldn24-GluA interaction

**Fig. 6.**
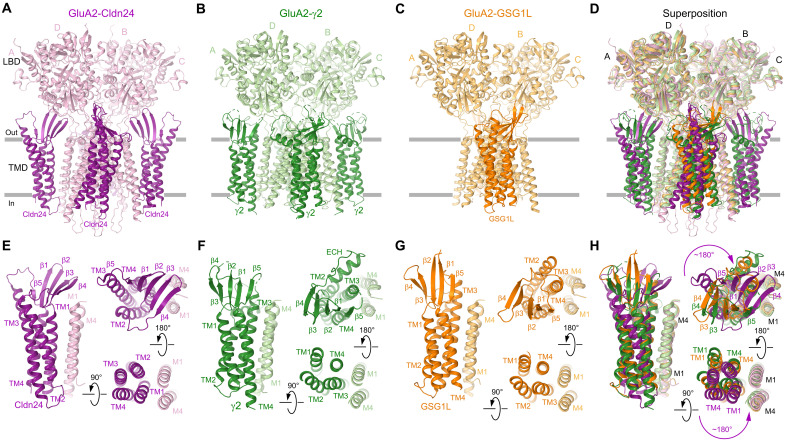
Structural prediction indicates potential binding modalities of auxiliary subunits with the GluA2 receptor. (**A**) AlphaFold3 predicted structure of GluA2 bound to Cldn24 viewed parallel to the membrane, with GluA2 core subunits colored pink and Cldn24 colored purple. (**B**) GluA2-γ2 [Protein Data Bank (PDB) ID: 7TNJ] viewed parallel to the membrane, with core GluA2 subunits colored in pale green and γ2 in dark green. (**C**) GluA2-GSG1L (PDB ID: 7RZ9) viewed parallel to the membrane, with core GluA2 subunits in pale orange and GSG1L in dark orange. (**D**) Superposition of GluA2-Cldn24, GluA2-γ2, and GluA2-GSG1L. (**E** to **H**) Close-up views of Cldn24 (E), γ2 (F), GSG1L (G), and their superposition (H), indicating an ~180° rotation of Cldn24 with respect to the TARP-γ2 and GSG1L.

Given the peculiar and circumscribed action of Cldn24 on GluAs compared to the well-known 4-TMD AMPAR auxiliary subunits of the TARP family and GSG1L, a closer look at its structural interaction with GluAs may be informative. In the absence of resolved structures of most claudins, we exploited the open-source artificial intelligence (AI) structure prediction tool AlphaFold3 (*27*, *28*). In Fig. 6, the predicted structure of Cldn24 and previously solved structures of the TARP-γ2 and GSG1L are shown.

The predicted structure of homomeric GluA2 in complex with Cldn24 displays four Cldn24 protomers binding to the tetrameric receptor, similar to that of the TARP-γ2 (Fig. 6, A and B). Unexpectedly, despite similar architecture of transmembrane and extracellular domains, Cldn24 is predicted to bind differently than TARP-γ2, specifically with a near 180° rotation compared to that of the TARP or GSG1L (Fig. 6H). This predicted rotation may explain the differences in affinity and kinetic modulation between Cldn24, TARPs, and GSG1L. Given the overall twofold symmetry of homomeric GluA2, interactions vary between A/C and B/D GluA2 subunits. Extensive interactions are predicted to occur between Cldn24 and subunit B of GluA2 (fig. S5D). The transmembrane domain 1 (TM1) of Cldn24 makes few hydrophobic interactions with the M4 of GluA2.

Besides hydrophobic interaction between transmembrane domains, it is generally assumed that the extracellular loops of both GSG1L and the TARPs may interact with the ligand-binding domains (LBDs) of GluAs or within their direct vicinity for gating modulation (*29*, *30*). Notably, different from GSG1L and the TARP-γ2, Cldn24 has markedly shorter extracellular loops (Fig. 6H) (*31*, *32*). As expected, interactions between the LBDs and the extracellular domain of Cldn24 are therefore minimal. However, electrostatic interactions are predicted to occur between the β1-β2 loop of Cldn24 and the GluA2 LBD lower lobe and the LBD-M4 linker, as well as the β3-β4 loop and the LBD-M1 linker (fig. S5, F and G). A neighboring Cldn24 protomer interacts with the GluA2 M1 through hydrophobic interactions with the TM1 helix of Cldn24. While interactions with the transmembrane helices are similar between Cldn24 and the A/C subunits of GluA2, much fewer electrostatic interactions are predicted with the LBD. Instead, the β1-β2 and β3-β4 loops of Cldn24 are predicted to primarily interact with loops in the lower lobe of the LBD and the LBD-M1 linker. Thus, AI modeling predicts that Cldn24 may represent a third claudin family member, besides the TARP-γ2 and GSG1L, regulating GluA function by structurally and allosterically distinct mechanisms (*29*).

## DISCUSSION

In this study, we identify Cldn24 as a previously unrecognized auxiliary subunit of AMPARs in mammalian CGCs. By speeding up the recovery of GluA receptors from desensitization, Cldn24 increases their availability after use, thereby enhancing their fidelity and changing their frequency response timing. Given its evolutionary origin as a scaffold for cell-to-cell junctions, we hypothesize that certain claudin family members may not only enlarge the functional diversity of AMPARs but also contribute to their subcellular localization. This finding sets a precedent whereby other proteins that reside in large diverse families may have unexpected effects in specific cell types.

Recent technical advances in proteomic analyses have allowed detailed characterization of the molecular composition of native ion channel complexes. For AMPARs, such studies have identified a large set of either transiently interacting proteins or integral complex constituents modulating AMPAR trafficking and biophysical gating (*33*–*37*). Moreover, smart use of mouse genetics and sophisticated protein biochemistry has provided exciting insight into AMPAR biogenesis (*1*, *38*, *39*). Despite the advantage of being unbiased, such proteomic approaches suffer from the limitation that lowly expressed or weakly interacting constituents of complexes may be overlooked. In the present study, we extensively screened the claudin family of proteins for putative action on GluA gating (*19*). Out of 19 claudin isoforms cloned from rat brain, Cldn24 emerged as a promising candidate for a previously unrecognized AMPAR auxiliary subunit. Its region-specific expression pattern in the CNS and the low detergent stability of its molecular interaction with GluAs, surviving only mild nonionic agents, may have led to underrepresentation in brain membrane solubilisates and kept it below the detection limits of combined affinity-purification and high-resolution tandem mass spectrometry analyses in earlier studies (*35*, *40*).

In the recombinant expression system of *X. laevis* oocytes, Cldn24 increased the amount of GluA receptors on the cell surface and modulated their gating properties. These effects were specific for glutamate receptors of the AMPA subtype, with no detectable effects on NMDA and KA subtype receptors, suggesting that Cldn24 may qualify as a previously unrecognized AMPAR auxiliary subunit. In situ hybridization revealed a distinct expression pattern of Cldn24 in the mouse CNS. Cldn24 mRNA was almost exclusively detected in granule cell layers of the olfactory bulb, the hippocampal formation, and, most prominently, the cerebellum. In these brain areas, all combinations of the pore-lining subunits GluA1, GluA2, and GluA4 prevail (*24*, *41*–*43*), which we showed are amenable to gating modulation by Cldn24. For the sake of completeness, it should be noted that GluA3 was not tested for modulation by Cldn24. Based on the assumption that Cldn24 integrates into postsynaptic AMPARs, as strongly indicated by our colocalization experiments in overexpressing hippocampal neurons and CGCs, we investigated whether native AMPARs in the latter cells contain receptor function-modulating Cldn24. Glutamate-induced currents in somatic outside-out patches from CGCs recovered from desensitization more slowly when Cldn24 was knocked out. Thus, Cldn24 represents a constituent of native AMPARs in CGCs, modifying their signaling properties.

To predict the architecture of the GluA-Cldn24 interaction, we used AlphaFold revealing a notable 180° rotation when compared to TARP-γ2 or GSG1L. This predicted structure resembles one possible conformation across a dynamic continuum of Cldn24 binding to the homomeric GluA receptor. However, the influence that the core receptor subunit assembly holds may further illuminate the most stable, long-lasting complex composition. Of note, the affinities for different auxiliary subunits, including TARPs, CNIHs, and now Cldn24, may vary, marked by the studies of calcium-permeable GluA2-containing AMPARs that showed selectively increased influence of TARP-γ2 (*44*). Given our functional studies on GluA2/4 heteromers, one may expect preferential association of TARP-γ2 with GluA2, and Cldn24 with GluA4.

CGCs receive one of the main inputs to the cerebellum conveyed by mossy fibers that signal in a highly divergent manner. They form several collaterals with numerous presynaptic boutons along the axon, each of them contacting >10 CGCs (*45*). Transmission at these synapses occurs at extraordinarily high frequencies up to 1.2 kHz while still being very efficient (*46*–*48*). A specialized presynaptic ion channel fitting allows for action potential generation and transmitter release at the required high temporal precision (*49*). For high-fidelity signaling, also the postsynapse must be equipped for that task, i.e., postsynaptic receptors must exhibit ultrafast gating properties (*50*). The combination of GluA2 and GluA4 in their alternatively spliced flop versions is the fastest at the level of the pore-lining subunits and predominantly expressed in CGCs (*24*). They are known to coassemble with the TARP-γ2 and GSG1L, which increase charge transfer by slowing deactivation and desensitization (*26*, *40*, *51*, *52*). However, both auxiliary subunits act at the expense of receptor fidelity, because they also delay their recovery from desensitization and hence limit their availability after use. Based on our results, we propose that Cldn24 corrects for this drawback and preserves synaptic fidelity by tuning the frequency response range to higher levels. A combination of electrophysiological and genetic experiments in native preparations will now be required to further dissect the functional role of Cldn24 in cerebellar high-frequency input signaling in concert with the other known AMPAR auxiliary subunits.

## MATERIALS AND METHODS

### Molecular biology

Whole brain RNA from *Rattus norvegicus* was isolated using the GeneElute Mammalian Total RNA Miniprep Kit (Sigma-Aldrich, Taufkirchen, Germany). The isolated RNA was used as a template to generate a cDNA library with SuperScript II reverse transcriptase (Thermo Fisher Scientific, Waltham, MA, USA), from which claudin constructs were cloned by polymerase chain reaction. All cDNAs were verified by sequencing. GenBank accession numbers of the clones used are BC061992.1 (Cldn1), NM_001106846.2 (Cldn2), NM_031700.2 (Cldn3), NM_001012022.1 (Cldn4), NM_031701.2 (Cldn5), NM_001102364.1 (Cldn6), NM_001037774.1 (Cldn8), NM_001011889.2 (Cldn9), NM_001106058.1 (Cldn10), NM_053457.3 (Cldn11), NM_001100813.1 (Cldn12), NM_001013429.1 (Cldn14), NM_001107135.3 (Cldn15), NM_001107112.1 (Cldn17), NM_001008514.1 (Cldn19), NM_001109394.1 (Cldn20), NM_001110143.2 (Cldn22), NM_001110144.1 (Cldn24), NM_001134638.1 (Cldn26), M38060.1 (GluA1_flip_), AF164344.1 (GluA2_flip_), M85035.1 (GluA2_flop_) NM_001113184.1 (GluA4_flop_), NM_019309.2 (GluK2), NM_017010.2 (GluN1–1a), NM_012574.1 (GluN2B), and AF093268.2 (Homer-1c). GluA2 was used in its native, Q/R-edited form with an arginine at the Q/R site, and GluK2 was used in its Q/R-unedited form. cDNAs were subcloned into the pSGEM vector or pMH4 for expression in *X. laevis* oocytes and neurons, respectively.

### cRNA preparation and heterologous expression in *Xenopus* oocytes

Prior to in vitro transcription, cDNA plasmids were linearized with Pac I or Nhe I. cRNA was synthesized from linearized cDNA templates using the mMESSAGE mMACHINE T7 Transcription Kit (Thermo Fisher Scientific). The quality of synthesized cRNA was checked by 1% agarose gel electrophoresis.

*X. laevis* oocytes were ordered from EcoCyte Bioscience (Dortmund, Germany). cRNAs were injected within 24 to 48 hours after surgery. For expression of homomeric receptors, 4 fmol or 8 fmol of the receptor subunit cRNA was injected, whereas for expression of heteromeric receptors, cRNAs were combined yielding a total amount of 4 or 8 fmol, respectively. If cRNAs coding for auxiliary subunits were coinjected, 2 or 4 fmol of each subunit cRNA was used, unless indicated otherwise.

### Cell culture of *Mus musculus* neurons

#### Hippocampal neurons

Animal experiments were carried out according to the Directive 2010/63/EU of the European Parliament. Procedures were approved by the responsible animal ethics committee [State Agency for Nature, Environment, and Consumer Protection (LANUV), no. VG 633/24]. Hippocampal neurons were prepared from embryonic day 16 (E16) C57BL/6J mice. Pregnant mother animals were anesthetized by isoflurane. E16 embryos were quickly dissected, and the brain was carefully transferred to dissociation buffer (Hanks’ balanced salt solution supplemented with 1 mM Hepes, pH 7.3; Thermo Fisher Scientific). The hippocampi were isolated and further digested with 0.05% trypsin to dissociate the neurons. The cells were seeded onto poly-d-lysine–coated coverslips at a density of 150,000 cells per 12-well in neurobasal/fetal calf serum medium [neurobasal supplemented with 7.5% fetal bovine serum, penicillin/streptomycin (1 μg/ml), amphotericin (2.5 μg/ml), and 1 mM sodium pyruvate; Thermo Fisher Scientific] and kept at 37°C and 5% CO_2_. After 4 hours, cells were transferred to 12-well plates with astroglia feeder cells at the bottom in an upside-down manner (*53*). After 5 days in vitro, neurons were cotransfected with the following plasmids: GluA1(HA-ex)/pMH4, Cldn24(Myc-ex)/pMH4, and Homer-1c-EGFP/pcDNA3.1 using Lipofectamine 2000 (Thermo Fisher Scientific).

#### Cerebellar granule cells

CGCs were prepared from postnatal day 4 (P4)– to P6-old C57BL/6J mice. Mice were anesthetized using isoflurane and euthanized by decapitation. The cerebellum was then dissected from the brain. The following steps, including dissociation, seeding, and culturing of cells, were the same as described for the hippocampal neuron preparation, except that cells and tissue pieces larger than 40 μm in diameter were removed using a cell strainer before seeding. After 2 to 7 days in vitro, neurons were cotransfected for patch clamp recordings with a pCas-Guide CRISPR vector, containing the *CLDN24* gene target sequence GCAGGATTTCCAGAGCCCCA, and a linear donor DNA containing LoxP-EF1A-tGFP-P2A-Puro-LoxP [*CLDN24* Mouse Gene Knockout Kit (CRISPR), OriGene Technologies, Rockville, MD, USA] using Lipofectamine 2000 (Thermo Fisher Scientific). Control cells were either transfected with pEGFP-C1 or left native. For imaging, neurons were cotransfected with the following plasmids: GluA4(HA-ex)/pcDNA3.1, Cldn24(Myc-ex)/pMH4, and Homer-1c-EGFP/pcDNA3.1 using Lipofectamine 2000 (Thermo Fisher Scientific).

#### Astroglia feeder cells

Astroglia feeder cells were prepared from neocortices of E16 embryonic C57BL/6J mice. The preparation was performed using a Neural Tissue Dissociation Kit and a gentleMACS Dissociator (Miltenyi Biotec, Bergisch Gladbach, Germany). The cells were plated onto 75-cm^2^ flasks and cultured in glia medium [Dulbecco’s modified Eagle’s medium + Glutamax, supplemented with 10% horse serum, 1 mM sodium pyruvate, amphotericin (2.5 μg/ml), and penicillin/streptomycin (0.1 μg/ml)] at 37°C and 5% CO_2_. When cells were near confluence (normally after 2 to 3 weeks), the astroglia cells were harvested and seeded onto 12-well plates. Seventy-two hours before the preparation of primary hippocampal neurons, the glia medium of the feeder cultures was changed to neuronal culture medium [neurobasal medium supplemented with 2% B27 supplement, penicillin/streptomycin (1 μg/ml), amphotericin (2.5 μg/ml), and 1 mM sodium pyruvate; Thermo Fisher Scientific) for preconditioning.

### Coimmunoprecipitation

Experiments were performed 4 days after cRNA injection as described previously (*35*, *36*). In brief, crude membrane fractions of *Xenopus* oocytes were obtained by ultracentrifugation (125,000*g*, 25 min) and solubilized for 30 min in S-buffer (in millimolar: 83 tris-HCl, 2.5 EDTA, 10 NaCl, 1.6 digitonin, 1 iodoacetamide, and protease inhibitors) on ice. Following an additional step of ultracentrifugation (131,000*g*, 15 min), the supernatants were incubated with anti-V5 antibody–coupled Dynabeads (no.13202, Cell Signaling Technology, Danvers, MA, USA; Thermo Fisher Scientific) for 2 hours at 4°C. After brief washing, bound proteins were eluted with Laemmli buffer.

### SDS–polyacrylamide gel electrophoresis and Western blotting

Crude membrane fractions or fractions from coimmunoprecipiation experiments were separated on 12% SDS–polyacrylamide gel electrophoresis. After electroblotting onto a polyvinylidene difluoride membrane (Roth), Western analysis was performed using mouse anti-GluA1 (1:1000; no. MAB2263, Merck Millipore, Burlington, MA, USA), rabbit anti-GluA1 (no. AB1504, Merck Millipore), rabbit anti-GluA2 (no. AB1768-I, Merck Millipore), mouse anti-V5 (1:5000), and rabbit anti-β-actin antibody (1:10,000; no. ab8227, Abcam), followed by goat anti-mouse or goat anti-rabbit immunoglobulin G (IgG)–horseradish peroxidase (HRP) (no. ab205719, Abcam; no. A16110, Thermo Fisher Scientific). Signals were detected using ECL Prime (Amersham).

### Quantification of GluA surface expression in *Xenopus* oocytes

Extracellular HA tagging was performed as described previously (*36*). All steps were performed at 4°C with gentle shaking and in the absence of detergents. In brief, oocytes were pretreated with 1% bovine serum albumin in Barth’s solution for 30 min and incubated with primary anti-HA antibody (1:100; no. sc-7392, Santa Cruz) followed by incubation with HRP-conjugated goat anti-mouse secondary antibody (1:5.000; no. ab205719, Abcam). Immunoreactivity was detected by enzymatic turnover of SuperSignal ELISA (enzyme-linked immunosorbent assay) Femto Maximum Sensitivity Substrate (Thermo Fisher Scientific) and quantified in a GloMax 20/20 luminometry system (Promega).

### Immunocytochemistry and imaging of neurons

Hippocampal neurons were surface-stained at 21 days in vitro (DIV21), 16 days posttransfection, with a rat antibody against HA (1:40; no. 11867423001, Roche, Basel, Switzerland) and a rabbit antibody against Myc (1:40; no. C3956, Sigma-Aldrich) for 10 min at 37°C. Cells were fixed with 4% paraformaldehyde in 120 mM sucrose/phosphate-buffered saline, permeabilized with 0.25% Triton X-100, blocked with 10% normal goat serum, and stained with a chicken antibody against microtubule-associated protein 2 (MAP2) (1:1000; no. ab5392, Abcam). The following secondary antibodies were used: goat anti-rat IgG conjugated to Abberior Star Orange (1:500; no. STORANGE-1007-500UG, Abberior), goat anti-rabbit IgG conjugated to Abberior Star Red (1:500; no. STRED-1002-500UG, Abberior), goat anti-chicken conjugated with Alexa Fluor 405 (1:500; no. A48260, Thermo Fisher Scientific), and GFP-Booster Atto488 (1:500; no. gba488-100, ChromoTek GmbH, Martinsried, Germany). Coverslips were mounted onto microscope slides using ProLong Diamond Antifade Mountant (Thermo Fisher Scientific). Confocal image acquisition was performed on a Leica Stellaris 8 FALCON [Light Microscopy Technology Platform (LiMiTec), Core Facility for Microscopy and Imaging, Bielefeld University, Germany] using a 100×/1.4 oil objective (HC PL APO CS2 100×/1.40 OIL), with excitation at 638/582/500/405 using a white light plus diode laser. Stimulated emission depletion (STED) image acquisition was performed on a Leica Stellaris 8 STED microscope (Leica Microsystems CMS GmbH, Meyerhofstraße 1, 69117 Heidelberg, Germany) using a 100×/1.4 oil objective (HC PL APO CS2 100×/1.4 OIL), with excitation at 637/587/495/405 using a white light plus diode laser and STED at 775/589 nm. We further applied TauSTED for fluorescence lifetime-based enhancement of resolution and signal-to-noise ratio (*54*), together with TauSTED Xtend for deconvolution with the lifetime-based effective point spread function (PSF) (Application Note TauSTED Xtend_MC-0006806_01032024), followed by image processing using Fiji.

### In situ hybridization of mouse brain slices

Brains from adult C57BL/6 mice were fresh-frozen on dry ice, and horizontal 15-μm sections were cut on a cryostat (Leica Microsystems, Germany). In situ hybridization experiments were carried out as described previously (*55*) with one of the following radiolabeled oligodeoxyribonucleotide probes: *CLDN24*, 5′ATGGCTTTCATCTTCAGAACGGCCATGCAATCAGTAGGGCTTTCT-3′; 5′GGGACCTCGAGATCATGGTTCATACCTAGAAAATGGAACTGTACAGCC-3′.

The oligodeoxyribonucleotide probes were 3′-end-labeled with (a)-^33^P-dATP (Hartmann Analytic, Germany) using terminal deoxynucleotide transferase (Roche, Basel, Switzerland). Brain sections were then incubated overnight in the hybridization mix containing 4× saline-sodium citrate buffer (0.6 M NaCl and 0.06 M sodium citrate), 50% formamide, 10% dextran, and labeled oligodeoxyribonucleotide probes (1 pg/μl) at 42°C, and were subsequently washed at 56°C for 30 min, dehydrated, and exposed to Kodak R X-Omat AR film for 2 weeks. Control in situ hybridization experiments were performed by including a 10-fold excess of unlabeled (cold) probes in the hybridization mix to confirm signal specificity through competitive inhibition.

### Electrophysiology

#### *TEVC recording from* X. laevis *oocytes*

TEVC recording was done using a TEC-10CX amplifier (npi electronic GmbH, Tamm, Germany) controlled by Pulse software (HEKA, Germany). TEVCs were carried out 4 days after injection. Borosilicate capillaries were pulled to resistances of 0.1 to 1 MΩ and filled with 3 M KCl. Oocytes were clamped at a holding potential of −70 mV and constantly perfused with normal frog Ringer’s solution (in millimolar: 10 Hepes-NaOH, pH 7.2, 115 NaCl, 1.8 CaCl_2_, and 2.5 KCl). GluA steady-state currents were recorded by applying l-glutamate (300 μM; Sigma-Aldrich, Taufkirchen, Germany) for 1 min. To prevent the receptors from desensitization, TCM (600 μM; Sigma-Aldrich, Taufkirchen, Germany) was coapplied with the agonist.

#### *Patch clamp recording from* X. laevis *oocytes*

Patch clamp recordings were performed 4 to 5 days after injection using an EPC 10 USB Patch clamp amplifier controlled by the software Patchmaster (HEKA, Germany). Prior to pulling outside-out patches, the vitelline membrane of the oocyte was removed using forceps. To achieve fast solution exchanges, a theta-barrel capillary was mounted on a piezoelectric linear actuator (PI Physik Instrumente, Karsruhe, Germany). The piezoelectric actuator was connected to an HVPZT amplifier (PI Physik Instrumente, Karsruhe, Germany) and an LPBF-01GX filter (npi electronic GmbH, Tamm, Germany) and could be controlled by the EPC 10 amplifier and Patchmaster, respectively.

Patch electrodes were pulled from borosilicate capillaries to resistances of 1 to 3 MΩ. The external solution was composed of (in millimolar) 140 NaCl, 10 Hepes, and 2 MgCl_2_, with the pH adjusted to 7.4 by the addition of NaOH. External solution was filtered through 0.45-μm nylon filters under vacuum. Patch pipettes were filled with internal solution containing (in millimolar) 110 d-gluconic acid, 140 KCl, 10 Hepes, 5 EGTA, and 2 MgCl_2_ and were adjusted to pH 7.4 with KOH. The osmolality of both solutions was adjusted to 285 to 295 mosmol/kg using water or NaCl and KCl, respectively. Recordings were performed at room temperature, and the patches were clamped at a holding potential of −70 mV. GluA deactivation and desensitization were recorded by applying glutamate (10 mM) for 1 and 100 ms, respectively. Glutamate applications were repeated 30 to 100 times. For recovery from desensitization, a two-pulse protocol was used, according to which a 100-ms glutamate application (10 mM) was followed by a second 100-ms glutamate pulse applied at different time intervals. To test different concentrations, recordings were performed as described before, using 1 mM, 300 μM, 100 μM, 30 μM, and 10 μM glutamate. The current responses were sampled at 50 or 100 kHz using a 2.9-kHz low-pass filter.

#### Patch clamp recording from CGCs

Patch clamp recordings from CGCs were performed at DIV13 to 28, or 16 to 22 days posttransfection. The setup used for recordings was the same as described above. Transduced granule cells were selected by green fluorescent protein (GFP) fluorescence.

### Data analysis

The offline analysis of patch clamp recordings was done with Igor Pro 8.04 (Wavemetrics, Lake Oswego, USA). Traces were baseline-subtracted and averaged following a monoexponential fit to determine the time constant τ, as well as the 10 to 90% rise time. For single-channel properties, nonstationary noise analysis was used as previously described (*56*). A train of 72 to 108 short (1 ms) glutamate responses was divided into 100 bins and compared to its average trace. The single-channel current (*i*), the number of channels (*N*), and background variance $\sigma_{B}^{2}$ (noise) were determined by plotting the ensemble variance ($\sigma^{2}$) against the average current ($\bar{I}$) and fitting with a parabolic function

$$\sigma^{2}=i\bar{I}-\frac{\bar{I^{2}}}{N}+\sigma_{B}^{2}$$

The single-channel conductance can be calculated with the holding potential *V*_H_

$$\gamma =\frac{i}{V_{H}}$$

The peak open probability *P*_O_ can be calculated as the fraction of open ion channels at a current peak

$$P_{O}=\frac{I_{\text{peak}}}{N_{i}}$$

Recovery from desensitization was determined as described by Robert and Howe (*22*). The recorded traces from two-pulse protocols were pooled and normalized. Data were fit to a monoexponential Hodgkin-Huxley equation

$$I_{t}={\left[{I_{\text{max}}}^{1/m}-\left({I_{\text{max}}}^{1/m}-{I_{0}}^{1/m}\right)e^{\left(-t/\tau \right)}\right]}^{m}$$

*I_t_* is the peak current at a given interpulse interval (*t*), *I*_max_ is the peak current at long interpulse intervals, *I*_0_ is the current at *t* = 0, τ is the recovery time constant, and *m* describes the number of kinetically equivalent rate-limiting transitions within the recovery time course. In Fig. 1G, the Hodgkin-Huxley fit yielded an *m* value of 1.77 for GluA1 alone, consistent with previous results (*22*).

### Kinetic modeling

Ordinary differential equations of the previously developed kinetic scheme (*22*) for GluA1 were implemented and solved (ode15s) in MATLAB (MathWorks). We implemented the model and used the rate constants outlined by Coombs *et al.* (*21*) to simulate the observed experimental data. While we achieved reasonable comparisons for desensitization, recovery from desensitization, and dose-dependent steady-state currents, deactivation gating proved to be far slower compared to our experimental results. Consequently, we defined the new rate constants *k*_3_ and *k*_−3_, respectively, recognizing that deactivation and desensitization show distinct behaviors. Subsequently, we used the “fminsearch” function in MATLAB to identify the minimum of an unconstrained multivariable function. Through this approach, we derived a new set of rate constants that best aligned with our experimental data for GluA1 gating in the absence and presence of Cldn24.

### In silico structural modeling

Models for GluA2 in complex with Cldn24 were generated in silico using AlphaFold3 (v3.0.1) (*27*) based on protein sequences from UniProt accession numbers P19491 (GluA2) and D4A2B4 (Cldn24). For ease of representation and minimization of computational resources, predicted disordered domains of GluA2 and Cldn24 (V848-I883 of GluA2 and P191-V220 of Cldn24) and the N-terminal region of GluA2 (M1-G410) were removed. Predicted complexes were visualized in ChimeraX and PyMOL. Structural comparison was done using models for GluA2-γ2 [Protein Data Bank (PDB) ID: 7TNJ] and GluA2-GSG1L (PDB ID: 7RZ9).

### Statistics

Data are given as means ± SEM unless otherwise stated. *N* refers to the number of oocytes recorded in TEVC experiments (always from >2 different batches), whereas *n* refers to the number of recorded patches in patch-clamp experiments. Statistical analysis was performed with GraphPad Prism 8 (GraphPad Softwares, San Diego, USA). When analyzing one parameter per patch, we first checked whether the data were normally distributed using an *F* test or Shapiro-Wilk normality test. Normally distributed data were analyzed using an unpaired two-tailed Student’s *t* test or a parametric analysis of variance (ANOVA) following a Dunnet’s or Tukey’s multiple comparisons test. Non-normally distributed data were analyzed using a Mann-Whitney *U* test or a Kruskal-Wallis test with Dunn’s post hoc test. Asterisks indicate standard levels of statistical significance: *P < 0.05, **P < 0.01, and ***P < 0.001. When analyzing more than one parameter from data obtained from the same patch, data had to be corrected for family-wise error. To this end, α was divided by the number of parameters to get adjusted levels of statistical significance, indicated by hash symbols.
