# Supplementary material for: Claudin 24—A novel enhancer of AMPA receptor fidelity
Source: Sci Adv. 2026 Mar 6;12(10):eaeb0196. doi: 10.1126/sciadv.aeb0196 (PMC12965324; doi:10.1126/sciadv.aeb0196)
Supplement: Supplementary file 1 — Figs. S1 to S5 Legend for movie S1 [file sciadv.aeb0196_sm.pdf]

Science Advances
AAAS

Supplementary Materials for

Claudin 24—A novel enhancer of AMPA receptor fidelity

Tobias Strasdeit *et al.*

Corresponding author: Tobias Strasdeit, tobias.strasdeit@hhu.de; Michael Hollmann, michael.hollmann@rub.de; Nikolaj Klöcker, nikolaj.kloecker@uni-duesseldorf.de

Sci. Adv. **12**, eaeb0196 (2026)
DOI: 10.1126/sciadv.aeb0196

The PDF file includes:

Figs. S1 to S5
Legend for movie S1

Other Supplementary Material for this manuscript includes the following:

Movie S1

Supplementary Information

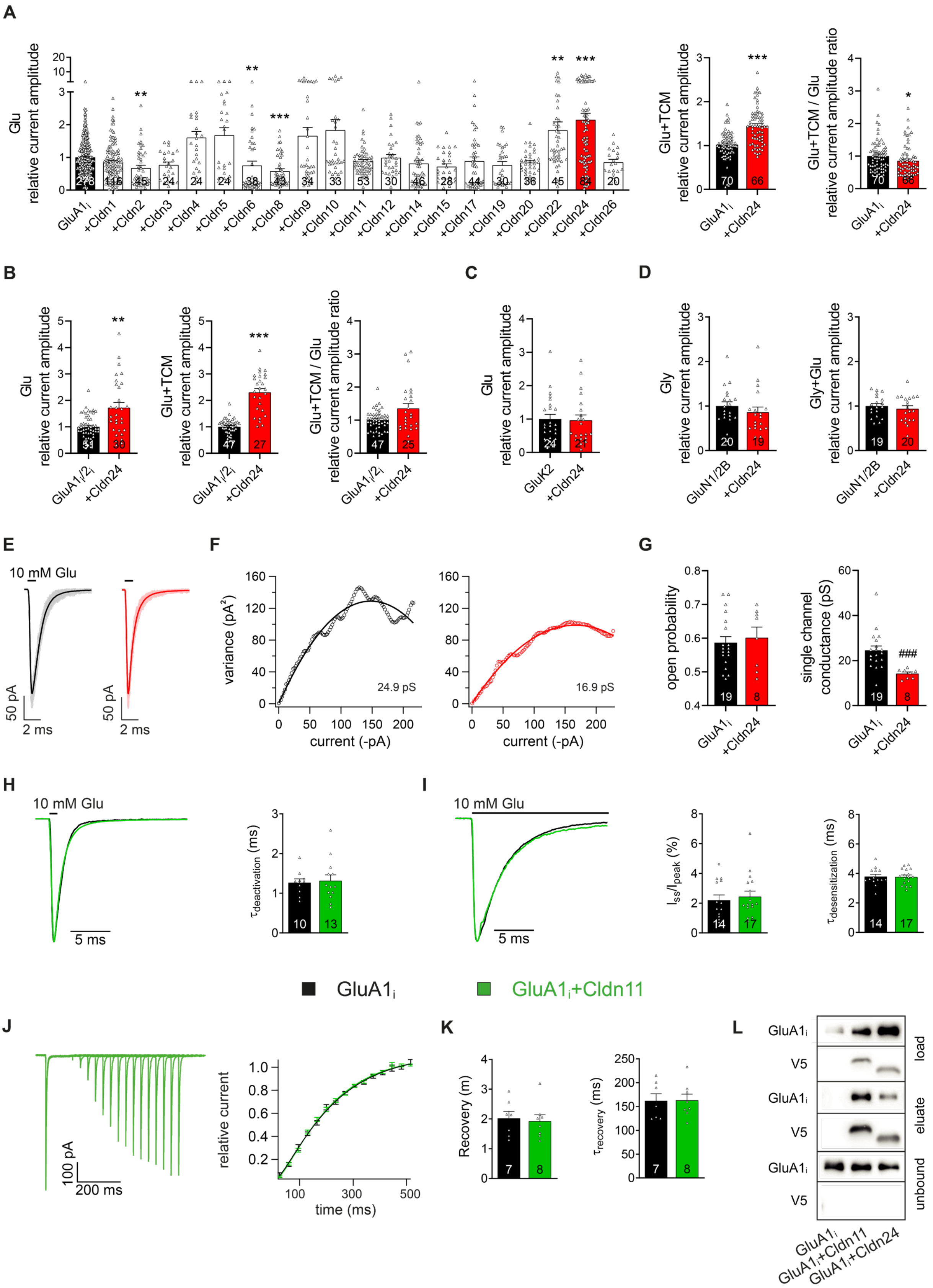

Figure S1.

A Quantification of normalized 300 µM glutamate-induced steady-state current responses recorded by TEVC from *Xenopus laevis* oocytes (± SEM) of GluA1 alone or co-expressed with all claudins cloned from brain (left). GluA1 current amplitudes (N = 278, SEM = 0.03) were more or less influenced by Cldn1 (0.86 ± 0.05fold, N = 116, p = 0.2697), Cldn2 (0.67 ± 0.09fold, N = 45, p = 0.0029), Cldn3 (0.77 ± 0.09fold, N = 24, p > 0.9999), Cldn4 (1.60 ± 0.19fold, N = 24, p = 0.1487), Cldn5 (1.68 ± 0.23fold, N = 24, p > 0.9999), Cldn6 (0.75 ± 0.14fold, N = 38, p = 0.0056), Cldn8 (0.58 ± 0.07fold, N = 43, p < 0.0001), Cldn9 (1.66 ± 0.26fold, N = 34, p > 0.9999), Cldn10 (1.84 ± 0.32fold, N = 33, p > 0.9999), Cldn11 (0.87 ± 0.06fold, N = 53, p > 0.9999), Cldn12 (0.99 ± 0.10fold, N = 30, p > 0.9999), Cldn14 (0.81 ± 0.10fold, N = 46, p = 0.1854), Cldn15 (0.72 ± 0.09fold, N = 28, p = 0.4010), Cldn17 (0.89 ± 0.13fold, N = 44, p = 0.3432), Cldn19 (0.76 ± 0.11fold, N = 30, p = 0.4278), Cldn20 (0.84 ± 0.07fold, N = 36, p > 0.9999), Cldn22 (1.83 ± 0.25fold, N = 45, p = 0.0072), Cldn24 (2.15 ± 0.20fold, N = 84, p < 0.0001), and Cldn26 (0.85 ± 0.09fold, N = 20, p > 0.9999). Quantification of normalized 300 µM glutamate/600 µM TCM-induced steady-state current responses of GluA1 alone or co-expressed with Cldn24 (middle). GluA1 current amplitudes in the presence of TCM (N = 70, SEM = 0.04) were significantly augmented by Cldn24 (1.45 ± 0.05fold, N = 66, p < 0.0001). Quantification of normalized ratios between glutamate/ TCM-induced and glutamate-induced current responses of GluA1 alone or co-expressed with Cldn24 (right). The desensitization inhibiting effect of TCM on GluA1 (N = 70, SEM = 0.06) was marginally lowered by Cldn24 (0.86 ± 0.06fold, N = 66, p = 0.0169). **B** TEVC recordings of GluA1/2 upon 300 µM glutamate (left) and 300 µM glutamate/ 600 µM TCM application (middle). Both, glutamate-induced current (N = 51, SEM = 0.06) and glutamate/ TCM-induced current (N = 47, SEM = 0.04) were significantly augmented by Cldn24 (1.73 ± 0.20fold, N = 30, p = 0.0018; 2.30 ± 0.15fold, N = 27, p < 0.0001). Quantification of normalized ratios between glutamate/ TCM-induced and glutamate-induced current revealed that the desensitization inhibiting effect of TCM on GluA1/2 (N = 47, SEM = 0.04) was not changed by Cldn24 (1.35 ± 0.15fold, N = 25, p = 0.1277). **C** TEVC recordings of GluK2 upon 300 µM glutamate application. GluK2 current (N = 24, SEM = 0.14) was not changed by Cldn24 (0.96 ± 0.16fold, N = 21, p = 0.8633). **D** TEVC recordings of GluN1/2B upon 10 µM glycine (left) and 10 µM glycine/ 300 µM glutamate application (right). Both, glycine-induced current (N = 20, SEM = 0.09) and glycine/ glutamate-induced current (N = 19, SEM = 0.06) were not changed by Cldn24 (0.86 ± 0.12fold, N = 19, p = 0.3555; 0.94 ± 0.07fold, N = 20, p = 0.4950). **E** Representative overlay of individual current responses (gray, light red) and their averages (black, red) of GluA1 (left) and GluA1+Cldn24 (right) recorded from outside-out patches upon 1 ms applications of 10 mM glutamate. **F** Current-variance relationships for patches illustrated in E, fitted by a parabolic function yielding mean single-channel currents. The respective single-channel conductance was calculated for the respective holding potential of -70 mV. **G** Quantification of the open probability P_O, calculated as the fraction of open ion channels at current peaks (left). P_O of GluA1 (P_O = 0.59 ± 0.02, n = 19) is unaffected by co-expression of Cldn24 (P_O = 0.60 ± 0.03, n = 8, p = 0.7448). Cldn24 coexpression reduced the mean single-channel conductance of GluA1 homomers from 24.5 ± 1.94 pS (n = 19) to 14.2 ± 0.73 pS (n = 8, p < 0.0001, right). **H-K** Outside-out patch recordings of GluA1. The $\tau_{deactivation}$ time (1.26 ± 0.10 ms, n = 10), current ratio I_{ss}/I_{peak} (2.20 ± 0.36 %, n = 14), $\tau_{desensitization}$ (3.78 ± 0.16 ms, n = 14), $\tau_{recovery}$ (162 ± 14.7 ms, n = 7) and 'm' value (2.02 ± 0.23, n = 7) of GluA1 were not changed by Cldn11 ($\tau_{deactivation}$ = 1.32 ± 0.15 ms, n = 13, p = 0.8193; I_{ss}/I_{peak} = 2.44 ± 0.36 %, n = 17, p = 0.7101; $\tau_{desensitization}$ = 3.77 ± 0.13 ms, n = 17, p = 0.8602; $\tau_{recovery}$ = 163 ± 12.9 ms, n = 8, p > 0.9999; m = 1.93 ± 0.21, n = 8, p = 0.6943). **L** Co-immunoprecipitation of GluA1 and V5-tagged Cldn11 or V5-tagged Cldn24 with the antibodies indicated to the left. Data in A (left) were analyzed by Kruskal-Wallis test following Dunn's multiple comparisons with control and data in A (middle, right) and B-D by Mann-Whitney *U* test. * = p < 0.05, ** = p < 0.01, *** = p < 0.001. Data in G ($^{\#\#\#}$ = p < 0.0005) and H, I, K were analyzed by Mann-Whitney *U* test and corrected for family-wise error.

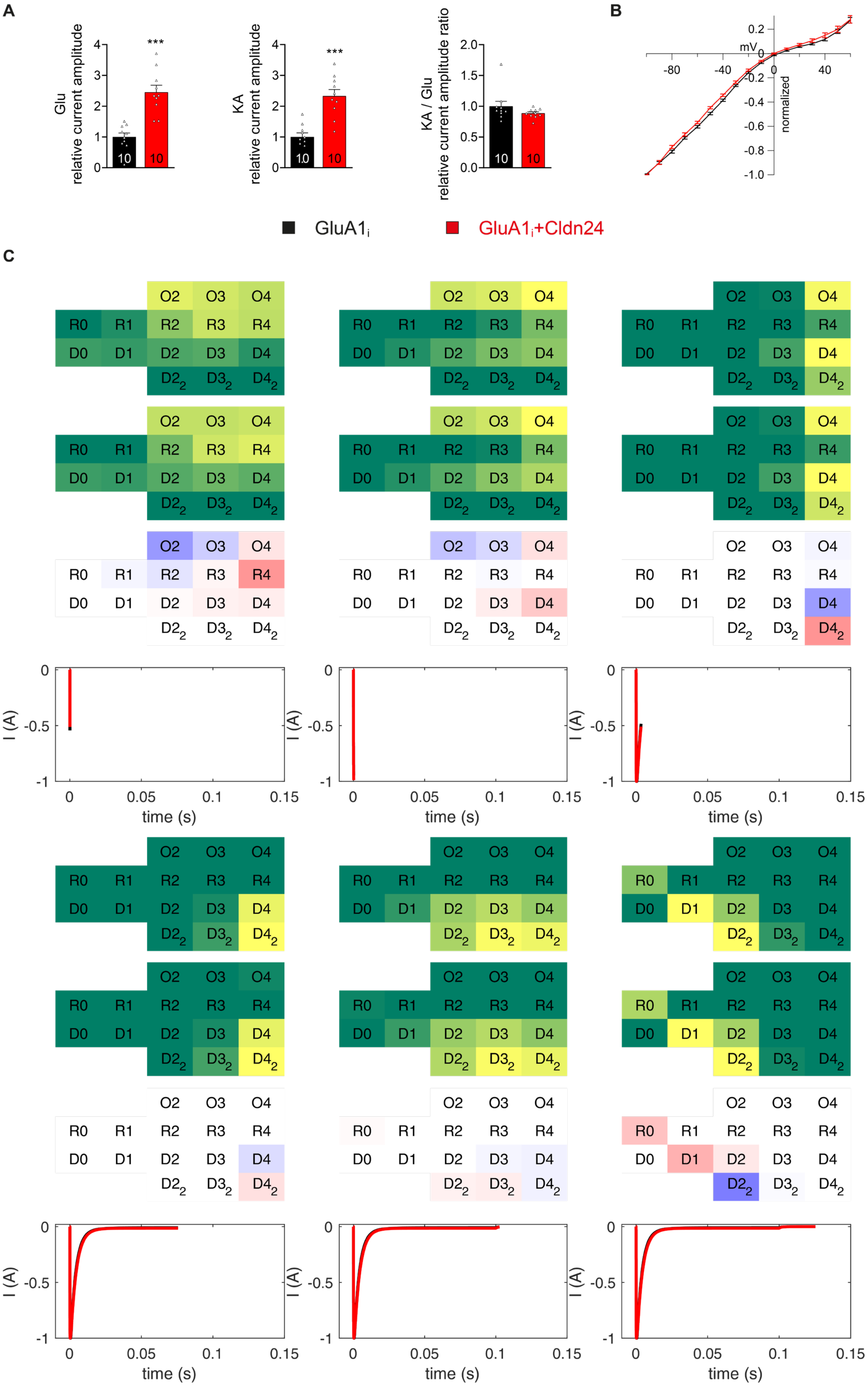

A
Glu
relative current amplitude
KA
relative current amplitude
KA / Glu
relative current amplitude ratio
***
***
10
B
mV
normalized
-80
-40
0
40
0.2
0
-0.2
-0.4
-0.6
-0.8
-1.0
GluA1i
GluA1i+Cldn24
C
O2 O3 O4
R0 R1 R2 R3 R4
D0 D1 D2 D3 D4
D2$_2$ D3$_2$ D4$_2$
I (A)
time (s)
0
-0.5
-1
0 0.05 0.1 0.15

Figure S2.

A Quantification of normalized 300 µM glutamate-induced steady-state current responses from *Xenopus laevis* oocytes expressing GluA1 alone or together with Cldn24 using TEVC (left). GluA1 current amplitudes (N = 10, SEM = 0.13) were significantly augmented by Cldn24 (2.46 ± 0.22fold, N = 10, p < 0.0001). Quantification of normalized 150 µM kainate-induced steady-state current responses of GluA1 alone and co-expressed with Cldn24 (middle). GluA1 current amplitudes (N = 10, SEM = 0.13) were significantly augmented by Cldn24 (2.33 ± 0.21fold, N = 10, p = 0.0001). Quantification of normalized ratios between kainate-induced and glutamate-induced current responses of GluA1 alone and co-expressed with Cldn24. The glutamate/ kainate ratio of GluA1 (N = 10, SEM = 0.08) was not changed by Cldn24 (0.89 ± 0.02fold, N = 10, p = 0.2475). **B** Current-voltage relationships of GluA1 alone or together with Cldn24 recorded from outside-out patches upon 100 ms applications of 10 mM glutamate. The shape of the current-voltage curve of GluA1 (n = 33) was not changed by Cldn24 (n = 21). **C** Snapshots of the different dwell time probabilities of the gating states at different time points of GluA1 and GluA1 co-assembled with Cldn24 and the relative changes by Cldn24, according to our kinetic model displayed in 2G. Dwell time probabilities from lower to higher probability are colored from green to yellow, and positive and negative changes in dwell times are gradually colored in red and blue, respectively. Data were analyzed by Mann-Whitney *U* test. *** = p < 0.001.

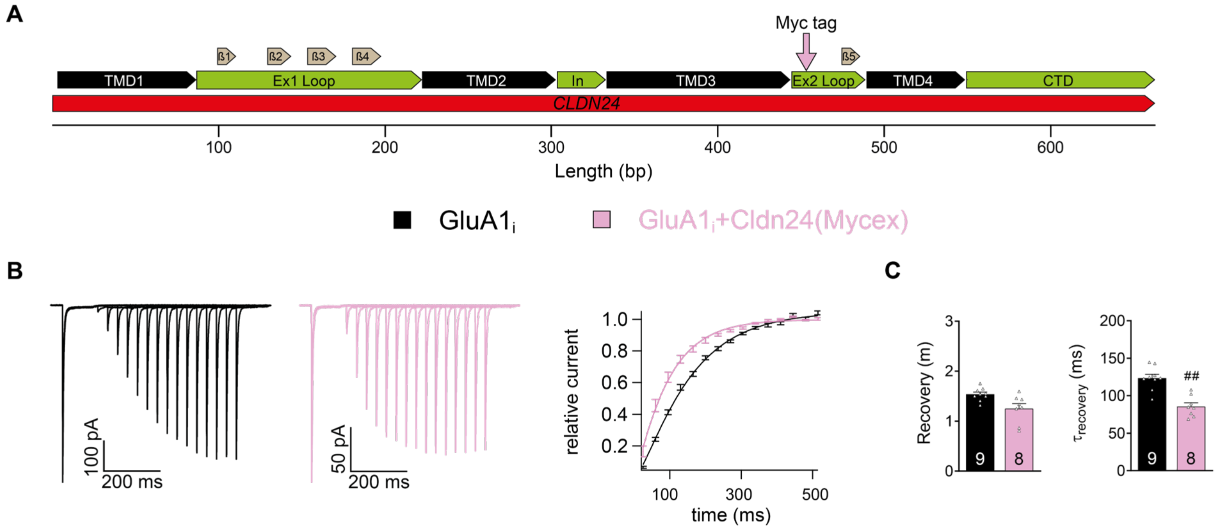

Figure S3.

A Schematic overview of Cldn24 domain structure and DNA sequence to demonstrate the cloning strategy of Myc-tag insertion. The Myc-tag was inserted into the second extracellular loop of Cldn24. **B** Recovery of GluA1 with and without Cldn24(Myc-ex). Recordings as in 1G. **C** Quantifications of 'm' and $\tau_{recovery}$, yielded by monoexponential Hodgkin-Huxley fits of the GluA1 recovery profiles. Recovery of GluA1 (123 ± 5.10 ms, n = 9) was significantly accelerated by the Myc-tagged Cldn24 (85.8 ± 4.89 ms, n = 8, p < 0.0006). The 'm' value of GluA1 (1.54 ± 0.04, n = 9) was marginally lowered by the Myc-tagged Cldn24 (1.25 ± 0.10, n = 8, p < 0.0152). Data were analyzed by Mann-Whitney *U* test and corrected for family-wise error. $^{\#\#}$ = p < 0.005.

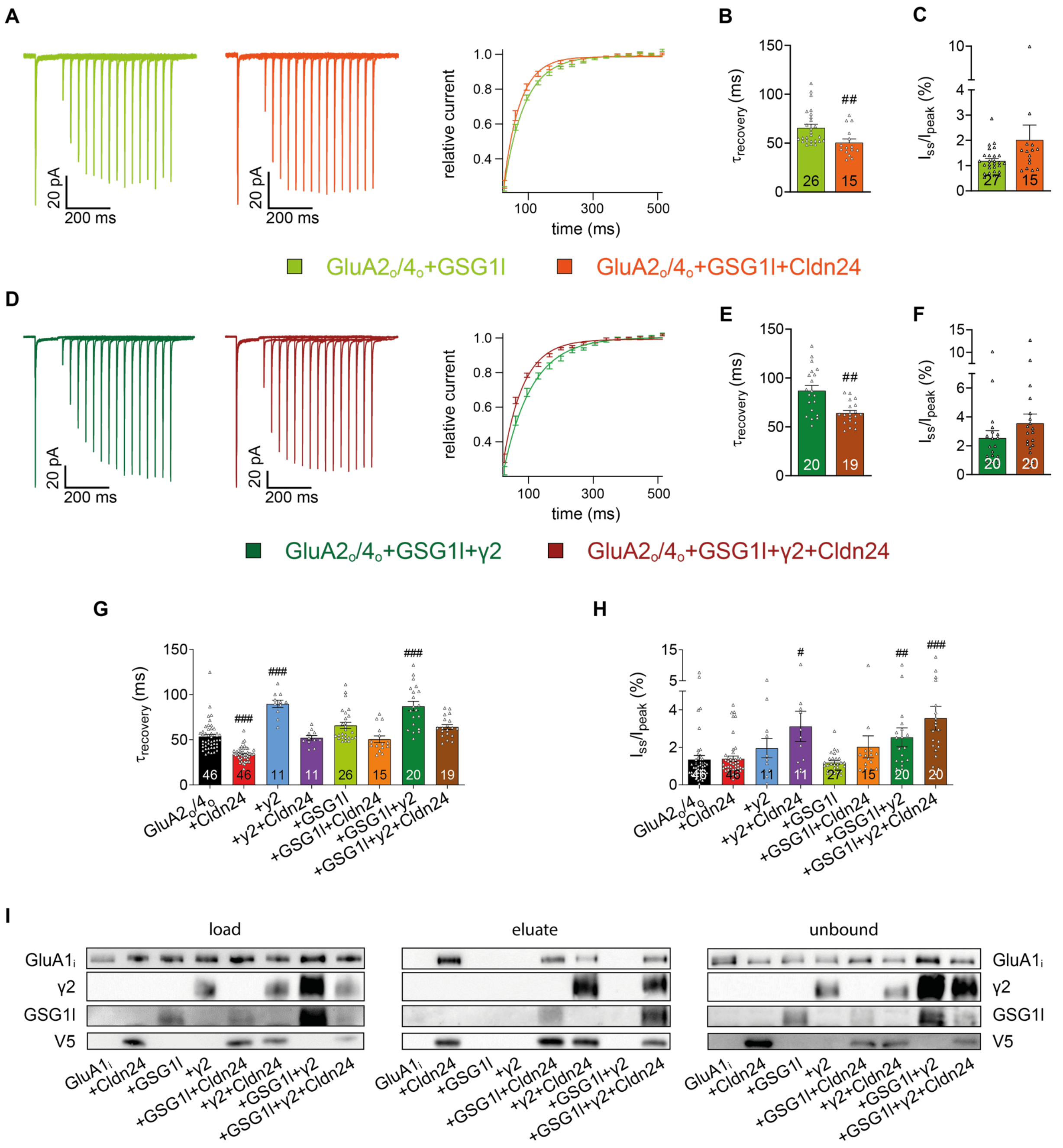

Figure S4.

A Recovery of GluA2/4 co-expressed with GSG1L with and without Cldn24. Recordings as in Figure 4A. **B** Recovery of GluA2/4 + GSG1l ($\tau_{recovery}$ = 65.8 ± 3.50 ms, n = 26) was significantly accelerated by Cldn24 ($\tau_{recovery}$ = 50.5 ± 3.72 ms, n = 15, p = 0.0014). **C** The current ratio I_{ss}/I_{peak} of GluA2/4 + GSG1l (1.19 ± 0.10 %, n = 27) tended to be increased by Cldn24 (2.02 ± 0.59 %, n = 15, p = 0.0698). **D** Recovery of GluA2/4 co-expressed with TARP γ2 and GSG1l with and without Cldn24. **E** Recovery of GluA2/4 + γ2 + GSG1l ($\tau_{recovery}$ = 87.2 ± 5.27 ms, n = 20) was significantly accelerated by Cldn24 ($\tau_{recovery}$ = 64.1 ± 2.68 ms, n = 19, p = 0.0021). **F** The current ratio I_{ss}/I_{peak} of GluA2/4 + γ2 + GSG1l (2.53 ± 0.50 %, n = 20) tended to be increased by Cldn24 (3.56 ± 0.64 %, n = 20, p = 0.1143). **G** Pooled $\tau_{recovery}$ of recovery profiles presented in Fig. 4B, E and fig S4B, E. Recovery of GluA2/4 was significantly accelerated by Cldn24 (p < 0.0001), but was significantly decelerated by γ2 (p = 0.0002) and by γ2 + GSG1l (p < 0.0001). Recovery of GluA2/4 remained unaltered by γ2 + Cldn24 (p > 0.9999), by GSG1l (p = 0.1042), by GSG1l + Cldn24 (p > 0.9999), and by GSG1l + γ2 + Cldn24 (p = 0.1327). **H** Pooled current ratios I_{ss}/I_{peak} presented in Fig.

4C, F and fig S4C, F. The current ratio I_{ss}/I_{peak} of GluA2/4 remained unaltered by γ2 ($p > 0.9999$), by GSG1l ($p > 0.9999$), and by GSGS1l + Cldn24 ($p = 0.1451$), but was significantly increased by γ2 + Cldn24 ($p = 0.0053$), by GSG1l + γ2 ($p = 0.0042$), and by GSG1l + γ2 + Cldn24 ($p < 0.0001$). **I** Co-immunoprecipitation of GluA1, V5-tagged Cldn24, GSG1L, and γ2 with the antibodies indicated to the left or right. Data were analyzed by Mann-Whitney *U* test (B, C, E, F), or Kruskal-Wallis test following Dunn's multiple comparisons with control (G, H) and corrected for family-wise error. $^{\#}$ = $p < 0.025$, $^{\#\#}$ = $p < 0.005$, $^{\#\#\#}$ = $p < 0.0005$.

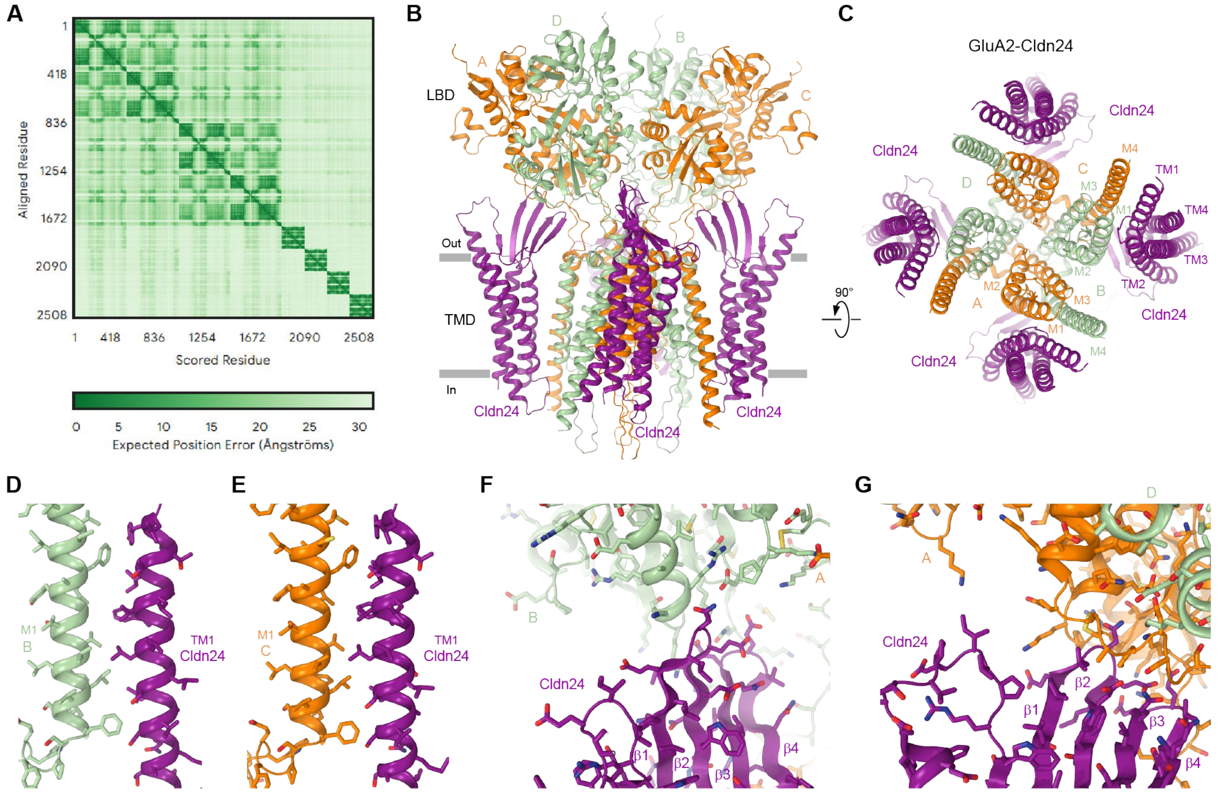

Figure S5.

A PAE plot featuring four copies of GluA2 LBD-TMD with 4x Cldn24. **B-C** AlphaFold3 predicted model of GluA2-Cldn24 viewed parallel to the membrane (**B**) or extracellularly (**C**), with GluA2 subunits A/C colored in orange, subunits B/D colored in pale green, and Cldn24 colored in purple. **D-E** Interactions between TM1 of Cldn24 with M1 of GluA2 subunit B (**D**) and M1 of GluA2 subunit C (**E**). **F-G** Interface between the extracellular head domain of Cldn24 and the lower lobe of LBD in GluA2 subunits B (**F**) and neighboring subunit A (**G**).

Movie S1. Dwell time probabilities over time of modeled gating states for GluA1 in the absence and presence of Cldn24. Dwell times of lower to higher probability are colored from green to yellow, whereas positive and negative changes in dwell times between the two test groups are gradually colored in red and blue, respectively.
